# Effects of Sea Animal Activities on Tundra Soil Denitrification and nirS‐ and nirK-Encoding Denitrifier Community in Maritime Antarctica

**DOI:** 10.3389/fmicb.2020.573302

**Published:** 2020-10-09

**Authors:** Hai-Tao Dai, Ren-Bin Zhu, Bo-Wen Sun, Chen-Shuai Che, Li-Jun Hou

**Affiliations:** ^1^Anhui Province Key Laboratory of Polar Environment and Global Change, School of Earth and Space Sciences, University of Science and Technology of China, Hefei, China; ^2^State Key Laboratory of Estuarine and Coastal Research, East China Normal University, Shanghai, China

**Keywords:** denitrification, nirS and nirK genes, quantitative PCR, community structure, tundra soils, maritime Antarctica

## Abstract

In maritime Antarctica, sea animals, such as penguins or seals, provide a large amount of external nitrogen input into tundra soils, which greatly impact nitrogen cycle in tundra ecosystems. Denitrification, which is closely related with the denitrifiers, is a key step in nitrogen cycle. However, effects of sea animal activities on tundra soil denitrification and denitrifier community structures still have received little attention. Here, the abundance, activity, and diversity of nirS‐ and nirK-encoding denitrifiers were investigated in penguin and seal colonies, and animal-lacking tundra in maritime Antarctica. Sea animal activities increased the abundances of nirS and nirK genes, and the abundances of nirS genes were significantly higher than those of nirK genes (*p* < 0.05) in all tundra soils. Soil denitrification rates were significantly higher (*p* < 0.05) in animal colonies than in animal-lacking tundra, and they were significantly positively correlated (*p* < 0.05) with nirS gene abundances instead of nirK gene abundances, indicating that nirS-encoding denitrifiers dominated the denitrification in tundra soils. The diversity of nirS-encoding denitrifiers was higher in animal colonies than in animal-lacking tundra, but the diversity of nirK-encoding denitrifiers was lower. Both the compositions of nirS‐ and nirK-encoding denitrifiers were similar in penguin or seal colony soils. Canonical correspondence analysis indicated that the community structures of nirS‐ and nirK-encoding denitrifiers were closely related to tundra soil biogeochemical processes associated with penguin or seal activities: the supply of nitrate and ammonium from penguin guano or seal excreta, and low C:N ratios. In addition, the animal activity-induced vegetation presence or absence had an important effect on tundra soil denitrifier activities and nirK-encoding denitrifier diversities. This study significantly enhanced our understanding of the compositions and dynamics of denitrifier community in tundra ecosystems of maritime Antarctica.

## Introduction

Nitrogen (N) is an essential element for the biosynthesis of key cellular components, such as proteins and nucleic acids, in all organisms (Kuypers et al., 2018). Nitrogen can be converted into multiple chemical forms as it circulates among atmosphere, terrestrial, and marine ecosystems, and microbial nitrogen conversion plays an important role in the nitrogen cycle (Lee and Francis, 2017). Denitrification is one of major biological nitrogen loss processes from natural ecosystems to atmosphere, contributing more than 70% of nitrogen loss (Dalsgaard et al., 2012; Hou et al., 2013; Babbin et al., 2014; Zheng et al., 2015). The denitrification processes are catalyzed by diverse types of metabolic enzymes, and closely related with the denitrifying microorganisms (Baker et al., 2015). Therefore, the abundance, activity, and diversity of denitrifying microorganisms have become research hotspots in the environments.

Nitrite reductases (Nir) is the rate-limiting enzyme among the enzymes that catalyze the denitrification processes (Baker et al., 2015; Chen et al., 2017). Nir encoded by nirS or nirK is structurally different but functionally equivalent (Shrewsbury et al., 2016). The nirS and nirK have been investigated in a variety of environments, including ocean (Braker et al., 2000; Shi et al., 2019), estuarine (Zheng et al., 2015; Gao et al., 2016), river and bay (Huang et al., 2011; Lee and Francis, 2017), wetland (Priemé et al., 2002), glacier foreland and arctic tundra (Heylen et al., 2006; Palmer et al., 2012), and in the rhizosphere (Guyonnet et al., 2018; Achouak et al., 2019). The nirK is found to be far less abundant than nirS in many environments (Mosier and Francis, 2010; Francis et al., 2013; Smith et al., 2015). The abundance, distribution, and diversity of denitrifying genes in environments are affected by multiple environmental variables, such as temperature, pH, salinity, dissolved oxygen, organic matter, and dissolved inorganic nitrogen (NO_3_^−^, NO_2_^−^, and NH_4_^+^) (Cornwell et al., 2014; Zheng et al., 2015; Gao et al., 2016; Li et al., 2017). At present, the abundance, diversity, and distribution of denitrifying genes have been investigated in the Antarctic environment, mainly concentrating on soils of King Sejong Station and the Cape Burk area (Jung et al., 2011; Han, 2013), Antarctic Peninsula (Yergeau et al., 2007; Vero et al., 2019), and the McMurdo Dry Valley (Ward and Priscu, 1997), and microbial mats of King George Island (Alcántara-Hernández et al., 2014; Valdespino-Castillo et al., 2018). However, the information on the distribution of nirS‐ and nirK-encoding denitrifiers, and their major environmental drivers is still limited in tundra soils of maritime Antarctica.

It is well known that Antarctica has extreme climate conditions with strong winds, limited liquid water availability, and low nutrient contents (Alcántara-Hernández et al., 2014). In coastal Antarctica, the ice-free tundra areas are often colonized by sea animals, such as penguins and seals, and tundra vegetation such as mosses, lichens, and algae. Penguin colonies, tundra vegetation around, and their interactions form a special ornithogenic tundra ecosystem (Tatur et al., 1997; Tatur, 2002). The global seabird database indicates that 69 million pairs of penguins are distributed on Antarctica and the sub-Antarctic islands (Riddick et al., 2012). Penguins provide a large amount of external nitrogen input to their colony soils through direct input of their guano and atmosphere deposition through ammonia volatilization (Sun et al., 2000; Zhu et al., 2011; Riddick et al., 2012). The N and P cycles in the ornithogenic tundra ecosystems are significantly affected by the deposition of a large amount of penguin guano (Riddick et al., 2012; Zhu et al., 2013, 2015b), which is abundant in organic carbon, nitrogen, and phosphorus (Zhu et al., 2011; Otero et al., 2018). The degradation of uric acid, as the main N compound in penguin guano, through mineralization and ammonification, produces NH_3_ or NH_4_^+^, which is subsequently oxidized to nitrate through nitrification, and eventually converted to N_2_ through denitrification (Kuypers et al., 2018; Otero et al., 2018). In addition, nitrous oxide (N_2_O), as a strong greenhouse gas and stratospheric ozone depletion substance, can be produced during the denitrification in soils (Bothe, 2000; Zhu et al., 2013). It has been found that sea animals significantly increased tundra N_2_O emissions in coastal Antarctica (Zhu et al., 2011, 2013, 2015b; Bao et al., 2018). Furthermore, penguin and seal colonies have a significant impact on tundra soil bacterial community structure (Ma et al., 2013; Zhu et al., 2015a), and the abundances, community compositions, and activities of ammonia oxidation archaea (AOA) and bacteria (AOB) are closely related to sea animal activities (Wang Q. et al., 2019). Every summer, a large number of penguin and seal breed on the ice-free land along the coasts of Antarctica and surrounding islands. Therefore, it is of great significance to examine the effects of penguin or seal activities on denitrification for understanding the nitrogen cycle process in tundra ecosystems. However, effects of penguin or seal activities on the abundances, community compositions, and activities of the denitrifiers still have received little attention in tundra soils of maritime Antarctica.

In this study, the soils were collected from a penguin colony, a seal colony, the adjacent animal-lacking tundra, and the slurry experiments were conducted to investigate the denitrification rates of tundra soils. Real-time quantitative PCR (qPCR) and high-throughput sequencing were conducted to investigate the abundance and diversity of nirS‐ and nirK-encoding denitrifiers in different tundra soils. Our main objectives were (1) to examine potential denitrification rates in tundra soils; (2) to investigate the abundance, diversity, and community structures of nirS‐ and nirK-encoding denitrifiers; and (3) to determine effects of sea animal activities and environmental variables on the abundances, community compositions, and activities of the denitrifiers in tundra soils of maritime Antarctica.

## Materials and Methods

### Study Area

**Figure 1:**
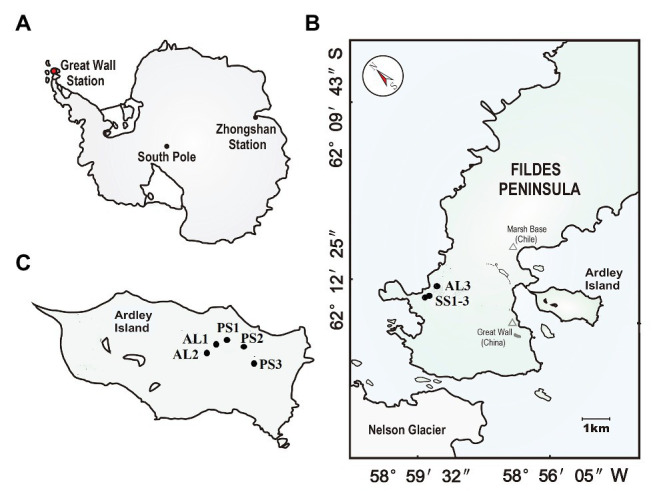
Soil sampling sites. **(A):** The red dot indicates the location of the study area in maritime Antarctica; **(B):** the location of the sampling sites on the Fildes Peninsula. The tundra soils included seal colony soils SS (SS1–SS3) in the western coast of the Fildes Peninsula and adjacent seal-lacking tundra soil AL3; **(C):** the location of the sampling sites on Ardley Island. The tundra soils included the eastern active penguin colony tundra soils PS (PS1–PS3) and the adjacent penguin-lacking tundra soils AL1 and AL2.

The study area is located on the Fildes Peninsula (61°51′–62°15′S, 57°30′–59°00′W) and Ardley Island (62°13′S, 58°56′W) in the southwest of King George Island (Figure 1). Fildes Peninsula is the largest ice-free area on King George Island in austral summer, covering an area of about 30 km^2^. It is one of the warmest and humidest areas in Western Antarctica due to effects of sub-Antarctic maritime climate. According to the long-term meteorological data collected at Great Wall Station on this peninsula, the mean annual temperature is about −2.5°C with a range of −26.6 ~ 11.7°C, and mean annual precipitation is 630 mm with the main form of snowfall.^1^ The lichens and mosses dominate local vegetation. On its western coast, there are some seal aggregations including elephant seal (*Mirounga leonine*), Weddell seal (*Leptonychotes weddellii*), fur seal (*Arctocephalus gazella*), and leopard seal (*Hydrurga leptonyx*) (Sun et al., 2004). During the breeding period every summer, a large amount of seal excreta is deposited into tundra soils by snowmelt water. In seal colonies, some seal wallows have been established due to strong seal activity, and tundra patches with sporadic vegetation have formed in the marginal zones of seal wallows, whereas the adjacent seal-lacking tundra areas are predominantly covered by mosses, lichens, and algae (85–90%) due to moderate soil fertility and the absence of seal tramp (Zhu et al., 2013).

Ardley Island, with about 2.0 km in length and 1.5 km in width, is connected to the Fildes Peninsula *via* a sand dam. This island is an important ecological reserve for penguins in Western Antarctica. The local prevailing wind direction is from west or northwest, which leads to the accumulation of less snow in the east of this island (i.e., the leeward slope), and allows the establishment of active penguin colonies mainly in the eastern coast. There are approximately 5,100 breeding pairs including Gentoo (*Pygoscelis papua*), Adélie (*Pygoscelis adeliae*), and Chinstrap penguins (*Phyllophora antarctica*) in the austral summer (Sun et al., 2004). In penguin colonies, many nesting sites and some small puddles are created by penguins. These nesting sites and puddles are highly enriched with penguin guano and devoid of vegetation due to toxic overmanuring and trampling. Many tundra patches with sporadic mosses, algae, and lichens have been formed around penguin nests and puddles. Ornithogenic Cryosols, rich in nitrogen and phosphorus, are well developed due to chemical weathering favored by penguin guano deposition and mineralization (Simas et al., 2007). The adjacent penguin-lacking tundra areas are almost completely (90–95%) covered by cushions of mosses, lichens, and algae. More detailed information about the study area had been given by Zhu et al. (2013).

### Tundra Soil Collection

In our study area, penguin and seal populations are spatially segregated, and penguin guano and seal excreta are transported by snowmelting water, respectively, and accumulated in local tundra soils or washed away in austral summer. During the period from December, 2018 to January, 2019, soil samples were collected from active seal colonies in the western coast of Fildes Peninsula, active penguin colonies in the east of Ardley Island and their adjacent animal-lacking tundra, to study effects of penguin and seal activities on tundra soil denitrification and nirS‐ and nirK-encoding denitrifier communities, although our soil sample numbers are limited due to local severe climatic conditions and the inaccessible areas. Over three soil samples PS1, PS2, and PS3 were collected from three sites of active penguin colonies, respectively, with the highest density and frequency of penguin populations during the breeding period. The two soil samples AL1 and AL2 were collected from two sites in adjacent penguin-lacking tundra, near the middle upland of Ardley Island, where penguins occasionally wander. In addition, three soil samples SS1, SS2, and SS3 were also collected at three sites of seal colonies, respectively, and one soil sample AL3 in adjacent seal-lacking tundra. All soils were collected from the top 5–10 cm using a clean stainless scoop. For each sampling site, triplicate sub-samples were collected, mixed, and homogenized to constitute a sample (about 300 g). After collection, each soil sample was divided into two parts: one part was stored at −80°C for microbial molecular analysis, and the other part was stored at −20°C for the analyses of soil physicochemical properties and denitrification activity.

### Analyses of Tundra Soil Physicochemical Properties

Soil samples were dried at 105°C to a constant weight to measure moisture content expressed as the percentage of weight lost. Organic matter (OM) was determined through the loss of ignition protocol, where soils were ignited in a muffle furnace for 4 h at 550°C after initial oven drying at 105°C. Soil pH was measured by mixing soil and Milli-Q water (1:2.5 ratio). Total nitrogen (TN), total carbon (TC), and total sulfur (TS) were measured using an elemental analyzer (vario MACRO, Elementar, Germany) (Zhu et al., 2011; Hou et al., 2015). The ammonium (NH_4_^+^-N) and nitrate (NO_3_^−^-N) were extracted from soils with 2 mol L^−1^ KCl, and measured using a continuous-flow nutrient analyzer (Skalar Analytical B.V., Netherlands) (Cheng et al., 2016). After digestion in Teflon tubes using HNO_3_-HCl-HF-HClO_4_ at 190°C, total phosphorus (TP) was measured using Inductively Coupled Plasma Optical Emission Spectrometer (ICP-OES; Perkin Elmer 2100DV, Waltham, MA, United States) (Gao et al., 2018).

### Determination of Denitrification Rates in Tundra Soils

The potential denitrification rates were determined using soil slurry experiments with a nitrogen isotope tracing method. Briefly, slurries were prepared with fresh soils and helium-purged ultrapure water at a soil/water volume ratio of 1:7. The slurries were transferred into a series of glass vials and pre-incubated for 48 h at 10°C, close to the highest air temperature (11.7°C) under summer collection conditions. After pre-incubated, the vials were spiked with helium-purged solutions of ^15^NO_3_^−^. The final concentration of ^15^N in each vial was approximately 100 μmol L^−1^. Half of the vials were immediately injected 200 μl 50% ZnCl_2_ solution into each vial to block slurry incubation. The remaining vials were incubated for 8 h at 10°C, and then were blocked by injecting 200 μl 50% ZnCl_2_ solution. The concentrations of ^29^N_2_ and ^30^N_2_ produced during the incubation were measured by membrane inlet mass spectrometry. The calculation of denitrification rates and more detailed information on slurry experiments have been described in the references (Hou et al., 2015; Cheng et al., 2016).

### DNA Extraction and High-Throughput Sequencing

Total DNA was extracted from 0.20 g soil with E.Z.N.ATM Mag-Bind Soil DNA Kit (OMEGA, United States). The extracted DNA was eluted in 60 μl of elution buffer. DNA integrity was checked by agarose gel electrophoresis (DYCZ-21, Beijing). The genomic DNA was quantified using Qubit2.0 DNA Assay Kit (Life, United States). The nirS gene fragment (425 bp) was amplified using primers Cd3aF (5'-GTSAACGTSAAGGARACSGG-3') and R3cdR (5'-GASTTCGGRTGSGTCTTGA-3') (Hallin and Lindgren, 1999; Yi et al., 2015). The nirK gene fragment (473 bp) was amplified using primers FlaCu (5'-ATCATGGTSCTGCCGCG-3') and R3Cu (5'-GCCTCGATCAGRTTGTGGTT-3') (Throbäck et al., 2004; Zheng et al., 2015). The 50 μl PCR mixtures contained 25 μl of *Taq* PCR Master Mix, 1 μl of template DNA, 2 μl of each primer (10 μM) and 20 μl of Nuclease-free ddH_2_O up to 50 μl (Sangon Biotech, Shanghai, China). The PCR condition was 2 min at 94°C, followed by 35 cycles of 94°C (30 s), 57°C (30 s), and 72°C (1 min) and a final step of 72°C (10 min) for both the nirS and nirK genes. The amplification products were sequenced using Illumina MiSeq in Sangon Company (Shanghai, China). Although primers Cd3aF:R3cdR and FlaCu:R3Cu set are not comprehensive enough to target all nirS-encoding and nirK-encoding denitrifiers, they can be used to compare the denitrifier diversity between soil samples (Throbäck et al., 2004; Blackwood et al., 2005).

### Real-Time Quantitative PCR

The abundances of nirS and nirK genes were determined in triplicate by qPCR using a LightCycler480 II Real-time PCR System (Rotkreuz, Switzerland). The primers were the same as those used in high-throughput sequencing. The standard curves showed strong correlations between the threshold cycle (Ct) and the lg values of gene copy numbers (*R*^2^ = 0.9994 for nirS; *R*^2^ = 0.9992 for nirK). The melt curve was checked, and the amplification efficiencies for nirS and nirK were 90.3 and 107.6%, respectively. The standard curves were used to calculate the abundance of nirS and nirK in tundra soils.

### Sequence Processing and Phylogenetic Analysis

The nirS and nirK genes sequences were processed using Quantitative Insights Into Microbial Ecology (QIIME) 2 Version:2019.07 (Bolyen et al., 2019). QIIME 2 plugins, including Cutadapt, DeMUX, and DADA2 were used to control sequence quality (Martin, 2011; Callahan et al., 2016). The sequences with 97% similarity were assigned to one operational taxonomic unit (OTU) by QIIME2 plugins, q2-vsearch Version:2019.07 (Rognes et al., 2016). The closest gene sequences in the NCBI database were obtained using NCBI BLASTn tools with a cutoff *E* value <1e^−6^ (Xiong, 2006; Pearson, 2013). Neighbor-Joining Trees was created by MEGA X program and the reliability of the tree topologies was estimated by performing 1,000 bootstrapping replicates.

### Statistical Analysis

The diversity indexes, including Chao 1, Shannon-Wiener (*H*), Simpson index (1/*D*), and Pielou’s evenness, and the abundance-based coverage estimate Ace were calculated by the R Version:3.6.1 (Shi et al., 2019). Chao 1 was used to estimate total OTU number for the sequences of nirS or nirK genes, Shannon-Wiener (*H*) and Simpson index (1/*D*) indicated alpha diversity for nirS‐ or nirK-encoding denitrifiers, and Pielou’s eveness represented the evenness of the denitrifier community in tundra soils. The abundance-based coverage was used to estimate the gene library coverage. One-way analysis of variation (ANOVA) and *T*-test were calculated for the comparisons between nirS, nirK gene abundances, diversity, and denitrification rates between tundra soils using SPSS Version:20. Correlations between nirS and nirK gene abundances, denitrification rates, and environmental factors were obtained by Pearson correlation analysis (Xiong et al., 2012). The relationships between denitrifying bacterial community structure and environmental factors were explored using canonical correspondence analysis (CCA) on the basis of the results of detrended correspondence analysis (DCA) in the software Canoco Version: 5.0 (Danovaro and Gambi, 2002).

## Results

### Physicochemical Properties of Tundra Soils

Soil physicochemical properties showed high heterogeneity across different types of tundra sites in maritime Antarctica (Table 1). Penguin colony soils (PS1, PS2, and PS3) and the adjacent penguin-lacking tundra soils (AL1 and AL2) were slightly acidic with the pH range from 5.3 to 6.2, whereas seal colony soils (SS1–SS3, AL3) were neutral to slightly alkaline with small pH variation (7.0–7.6). The highest levels of MC, OM, TN, TC, TS, TP, and NH_4_^+^-N occurred in penguin colony soils (PS1–PS3). Compared with animal-lacking tundra soils, active penguin and seal colony soils had much higher TN, TS, TP, and NH_4_^+^-N contents most likely due to the deposition of penguin guano or seal excreta. Especially, the NH_4_^+^-N and TS contents in penguin colony soils (means: 73.4 mg NH_4_^+^-N kg^−1^ and 4.3 mg S g^−1^) and seal colony soils (means: 62.0 mg NH_4_^+^-N kg^−1^ and 2.1 mg S g^−1^) were one to two orders of magnitude higher than those in animal-lacking tundra soils (means: 4.2 mg NH_4_^+^-N kg^−1^ and 0.5 mg S g^−1^). In addition, penguin colony soils (PS1–PS3) and its adjacent tundra soils AL1 had higher NO_3_^−^-N (1.0–12.0 mg kg^−1^) than seal colony soils (SS1–SS3) and animal-lackingtundra soils AL2 and AL3 (0.1–0.2 mg kg^−1^). Seal colony soils had lower C:N ratios (mean:5.2) than penguin colony soils (mean:8.6) and animal-lacking tundra soils (mean:11.5). Overall, the deposition of penguin guano or seal excreta altered local soil biogeochemical properties, leading to generally low C:N ratios and high TN, TS, TP, and NH_4_^+^-N contents in fauna-related tundra soils.

**Table 1:** Soil properties at tundra sites on Ardley Island and Fildes Peninsula in maritime Antarctica.

Sampling No.	pH	MC	OM	TN	TC	TS	TP	C:N	NH_4_^+^-N	NO_3_^−^-N
		(%)	(%)	(mg g^−1^)					(mg Kg^−1^)	
Seal colony soils										
SS1	7.0 ± 0.2	23.2	5.54	1.8 ± 0.5	11.1 ± 2.5	1.3 ± 0.2	1.3 ± 0.1	6.0	22.9 ± 5.8	0.1 ± 0.0
SS2	7.3 ± 0.1	28.8	9.09	6.6 ± 0.5	34.6 ± 2.9	2.6 ± 0.3	5.0 ± 0.2	5.3	77.7 ± 10.2	0.2 ± 0.1
SS3	7.6 ± 0.1	21.6	8.48	4.8 ± 0.3	21.1 ± 1.8	2.3 ± 0.2	3.6 ± 0.2	4.4	85.4 ± 11.1	0.1 ± 0.0
Mean	7.3 ± 0.3^a^	24.5^a^	7.70^a^	4.4 ± 2.1^a^	22.3 ± 10.4^a^	2.1 ± 0.6^ab^	3.3 ± 1.9^a^	5.2 ± 0.8^a^	62.0 ± 31.8^a^	0.1 ± 0.1^a^
Penguins colony soils										
PS1	5.4 ± 0.1	68.2	47.9	20.5 ± 0.2	194.7 ± 1.5	3.4 ± 0.2	23.7 ± 0.3	9.5	67.2 ± 14.0	1.5 ± 0.4
PS2	5.3 ± 0.2	69.8	43.7	27.5 ± 0.7	180.5 ± 2.8	6.7 ± 0.2	32.9 ± 0.3	6.6	119.5 ± 29.3	1.4 ± 0.5
PS3	5.5 ± 0.1	64.7	20.9	9.6 ± 0.5	92.0 ± 5.6	2.8 ± 0.3	12.5 ± 0.3	9.6	33.5 ± 6.0	0.2 ± 0.1
Mean	5.4 ± 0.2^b^	67.5^b^	37.5^b^	19.2 ± 7.8^b^	155.7 ± 48.3^b^	4.3 ± 1.8^a^	23.0 ± 10.2^b^	8.6 ± 1.7^ab^	73.4 ± 41.0^b^	1.0 ± 0.9^a^
Animal-lacking soils										
AL1	5.7 ± 0.2	46.01	15.2	4.3 ± 0.1	39.0 ± 1.0	0.9 ± 0	8.1 ± 0.2	9.1	9.7 ± 2.8	12.0 ± 2.2
AL2	6.2 ± 0.1	35.48	8.80	2.3 ± 0.1	22.1 ± 1.3	0.5 ± 0.3	5.7 ± 0.1	9.5	2.7 ± 0.8	0.2 ± 0.0
AL3	7.1 ± 0.1	25.02	9.05	0.2 ± 0.0	3.2 ± 0.2	0.1 ± 0.0	0.2 ± 0.0	16.0	0.1 ± 0.1	0.1 ± 0.0
Mean	6.3 ± 0.6^c^	35.50^a^	11.0^a^	2.3 ± 1.8^a^	21.4 ± 15.5^a^	0.5 ± 0.4^b^	4.7 ± 4.1^a^	11.5 ± 3.9^b^	4.2 ± 3.6^a^	4.1 ± 6.9^a^

MC, the moisture content of soil; ND indicated that the soil sample was not determined. For the measured soil properties of each tundra, tundra soil types with different letters (a, b, or c) are significantly different from one another (ANOVA, *p* < 0.05).

### Gene Abundances of nirS and nirK in Tundra Soils

**Figure 2:**
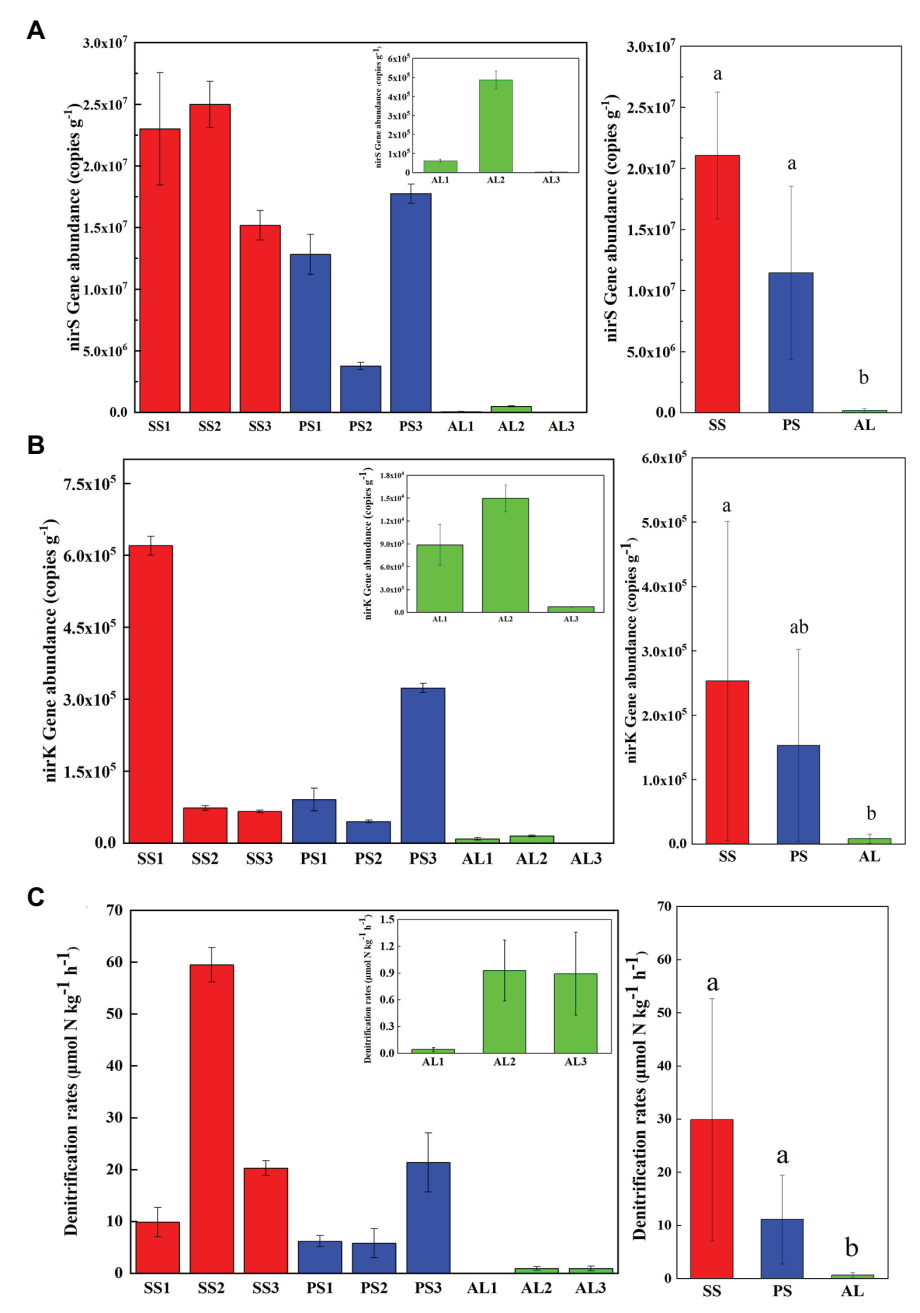
Comparisons of the abundances of nirS **(A)** and nirK **(B)** genes, and denitrification rates **(C)** in tundra soils of maritime Antarctica. Left figures presented the mean abundances of nirS **(A)** and nirK **(B)** genes, and the mean denitrification rates **(C)** in the soils of individual tundra site; Right figures indicated the mean abundances of nirS **(A)** and nirK **(B)** genes, and the mean denitrification rates in seal colony soils (SS), penguin colony soils (PS), and animal-lacking tundra soils (AL). The different letters indicated statistically significant differences (ANOVA, *p* < 0.05). The error bars indicate standard deviations of the averages.

**Figure 3:**
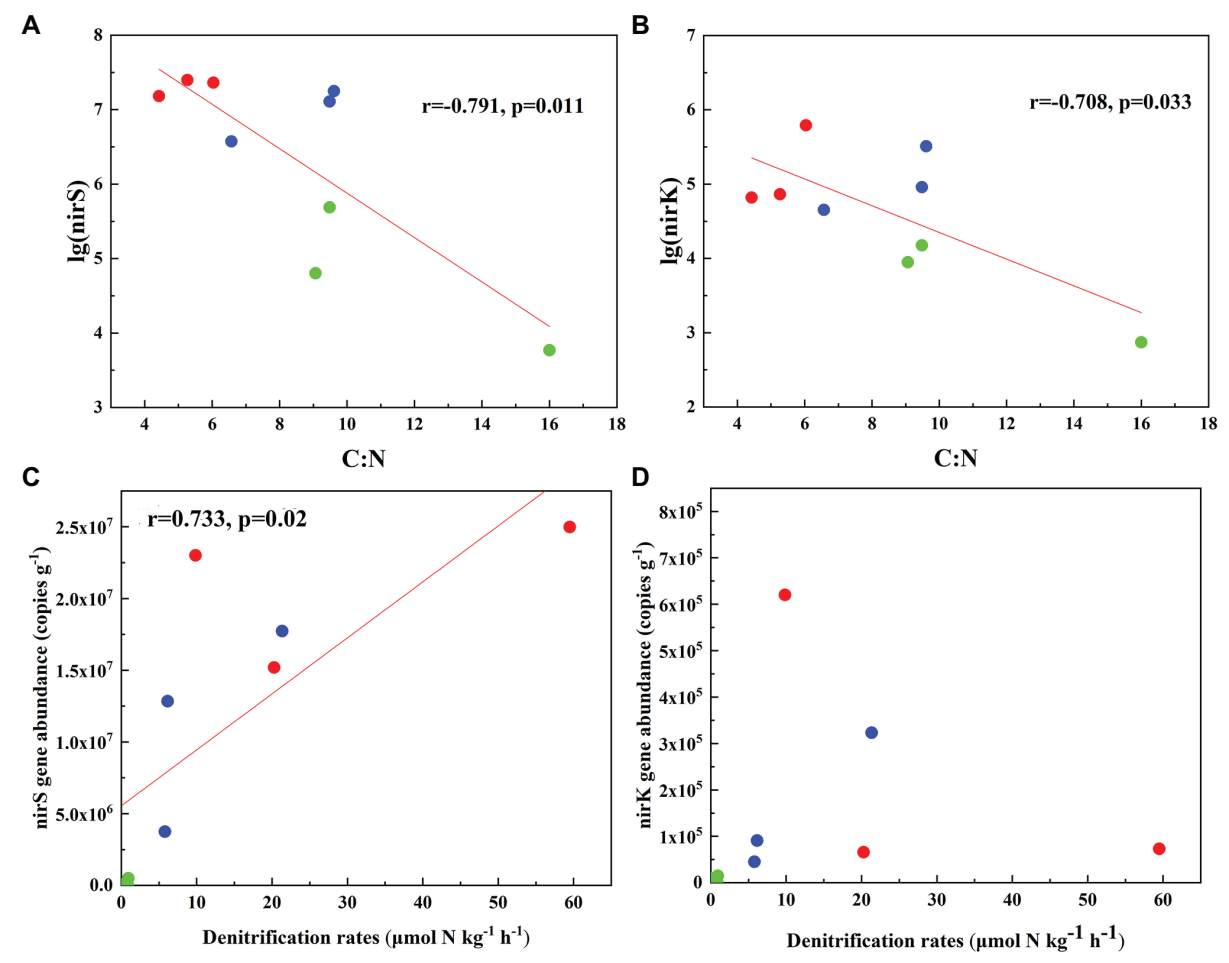
Pearson’s correlation between nirS and nirK genes abundances and tundra soil C:N **(A,B)**, and between denitrification rates and nirS, nirK gene abundances **(C,D)** in tundra soils of maritime Antarctica. The red, blue, and green dots represent the soils from SS, PS, and AL sites, respectively.

The abundances of nirS genes were significantly higher than those of nirK genes in all tundra soils (*t*-test, *n* = 9, *p* < 0.05). The abundances of nirS genes in penguin and seal colony soils (3.5 × 10^6^–2.5 × 10^7^ copies g^−1^) were two to four orders of magnitude higher than those in the animal-lacking tundra soils (5.9 × 10^3^–4.9 × 10^5^ copies g^−1^). The highest nirS gene abundance was detected in SS2, whereas the lowest occurred in AL3 (Figure 2A). The abundances of nirK genes showed heterogeneous distribution patterns among the tundra soils (Figure 2B). Overall, the abundances of nirK genes in PS (PS1–PS3) and SS (SS1–SS3) were higher than those in animal-lacking tundra soils AL (AL1–AL3). The gene abundances, especially for nirK genes, show a high variability even within the same sample types due to effects of the deposition of penguin guano or seal excreta, and levels of animal activities. The extremely high abundances of nirK genes occurred in SS1 (6.2 × 10^5^ copies g^−1^) and PS3 (3.2 × 10^5^ copies g^−1^), one to three orders of magnitude higher than those in other soils within animal colonies (4.5 × 10^4^–9.1 × 10^4^ copies g^−1^) and in adjacent animal-lacking tundra soils (7.4 × 10^2^–1.5 × 10^4^ copies g^−1^). The lg values of both nirS and nirK genes abundances showed significant negative correlations (*r* = −0.791, *p* = 0.011 for nirS; *r* = −0.708, *p* = 0.033 for nirK) with C:N ratios in tundra soils (Figures 3A,B). No significant correlation was obtained between nirS and nirK genes abundances and other environmental parameters (Supplementary Table S1). Therefore, tundra soil C:N ratios were predominant factors affecting nirS and nirK gene abundances in maritime Antarctica.

### Denitrification Rates in Tundra Soils

The heterogeneous distribution pattern for denitrification rates was observed among different tundra soils, with the range from 0.04 to 59.49 μmol N kg^−1^ h^−1^ (Figure 2C). Overall, the denitrification rates in penguin or seal colony soils were significantly higher (*p* < 0.05) than those in animal-lacking tundra soils. The mean highest rate occurred in seal colony soils (mean 29.88 ± 22.77 μmol N kg^−1^ h^−1^), followed by penguin colony soils (mean 11.10 ± 8.34 μmol 11 N kg^−1^ h^−1^), two orders of magnitude higher than those in animal-lacking tundra soils (mean 0.62 ± 0.50 μmol N kg^−1^ h^−1^). Of all tundra soils, the highest rate occurred at SS2 (59.49 ± 3.3 μmol N kg^−1^ h^−1^) with the highest nirS gene abundance. Even within penguin or seal colony, the denitrification rates also showed high variability between tundra soil samples due to effects of these animal activities and the deposition of their excreta. The denitrification rates in SS2, SS3, and PS3 (20.29–59.49 μmol N kg^−1^ h^−1^) were one order of magnitude higher than those in SS1, PS1, and PS2 (5.79–9.85 μmol N kg^−1^ h^−1^). The denitrification rates were significantly positively correlated with nirS gene abundances (*r* = 0.733, *p* = 0.02) in tundra soils, but no correlation was obtained between denitrification rates and nirK gene abundances (Figures 3C,D). In addition, no significant correlations were found between the denitrification rates and soil physicochemical properties in maritime Antarctic tundra (Supplementary Table S1).

### Diversity for nirS‐ and nirK-Encoding Denitrifiers in Tundra Soils

**Figure 4:**
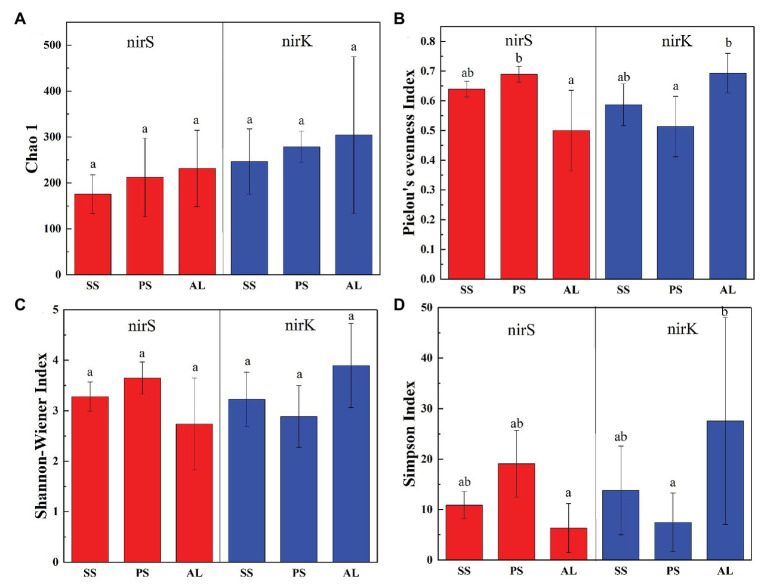
Richness and diversity of nirS and nirK genes in seal colony soils (SS), penguin colony soils (PS), and animal-lacking tundra soils (AL) in maritime Antarctica. **(A)** Chao 1, **(B)** Pielou’s evenness Index, **(C)** Shannon-Wiener Index, **(D)** Simpson Index. The error bars indicate standard deviations of the averages. The different letters indicated statistically significant differences (ANOVA, *p* < 0.05).

**Figure 5:**
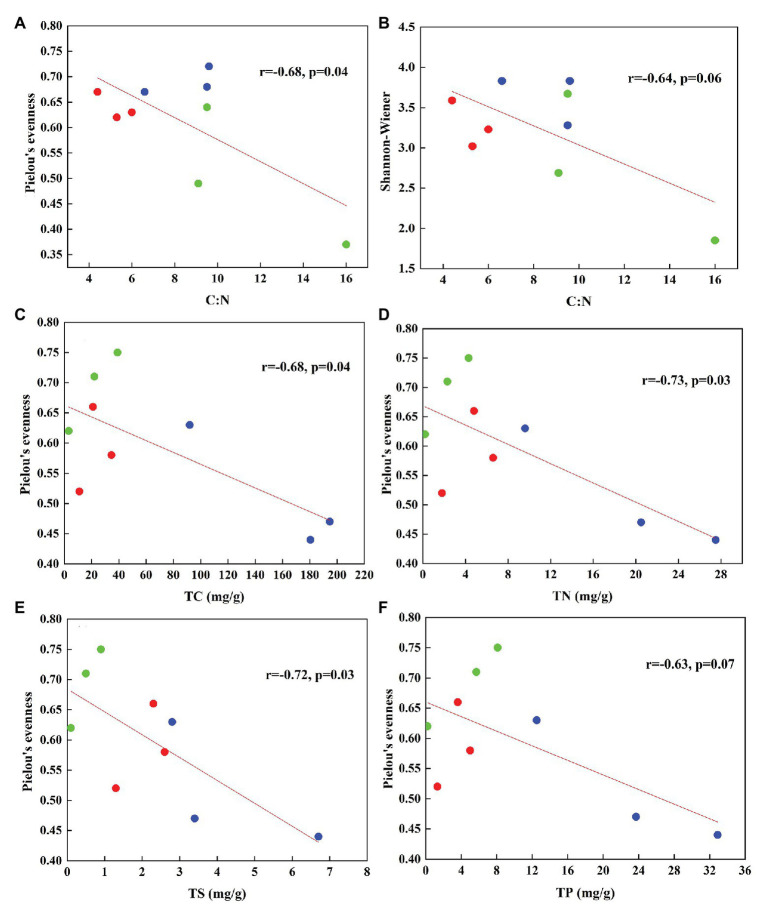
The relationships between the diversity of nirS type **(A,B)** and nirK type **(C–F)** denitrifiers and soil biogeochemical parameters associated with sea animal activities in tundra soils of maritime Antarctica (*n* = 9). The red, blue, and green dots represent the soils from SS, PS, and AL sites, respectively.

In total, 470,556 high-quality sequences of nirS genes were obtained from all tundra soils. These sequences were clustered into 1,095 OTUs with 97% similarity. As for nirK genes, 385,042 high-quality sequences were obtained and clustered into 1,692 OTUs with 97% similarity. The Chao 1 of nirK-encoding denitrifiers (120–458) was higher than that of nirS-encoding denitrifiers (128–304) (Supplementary Table S2). The Pielou’s evenness, Shannon-Wiener, and Simpson (1/*D*) indexes of nirK-encoding denitrifiers were higher than those of nirS-encoding denitrifiers in animal-lacking tundra soils (AL1, AL2, and AL3) but lower in sea animal colony soils. The three indexes for the nirS-encoding denitrifiers were higher in penguin or seal colony soils than in animal-lacking tundra soils (Figure 4). On the contrary, the three indexes of nirK-encoding denitrifiers were lower in animal colony soils than those in animal-lacking tundra soils. The Pielou’s evenness indexes for nirS‐ and nirK-encoding denitrifiers in penguin colony soils had significant difference (ANOVA, *p* < 0.05) from those in animal-lacking tundra soils. The Simpson (1/*D*) indexes for the nirK-encoding denitrifiers were significantly lower in penguin colony soils than in animal-lacking tundra soils (ANOVA, *p* < 0.05). For the same soil sample types, the Pielou’s Evenness Index and Shannon-Wiener Index (*H*) for the nirS‐ and nirK-encoding denitrifiers showed a low variability with the small standard deviations of the means. However, the Simpson Index (1/*D*) showed a high variability even between the same soil sample types (Supplementary Table S2). In addition, the Pielou’s evenness index of nirS-encoding denitrifiers showed significant negative relationships with C:N ratios (*R* = −0.68, *p* < 0.05), whereas the evenness index of nirK-encoding denitrifiers showed significant negative relationships with TN, TC, and TS (Figure 5).

### Compositions of nirS‐ and nirK-Encoding Denitrifiers in Tundra Soils

**Figure 6:**
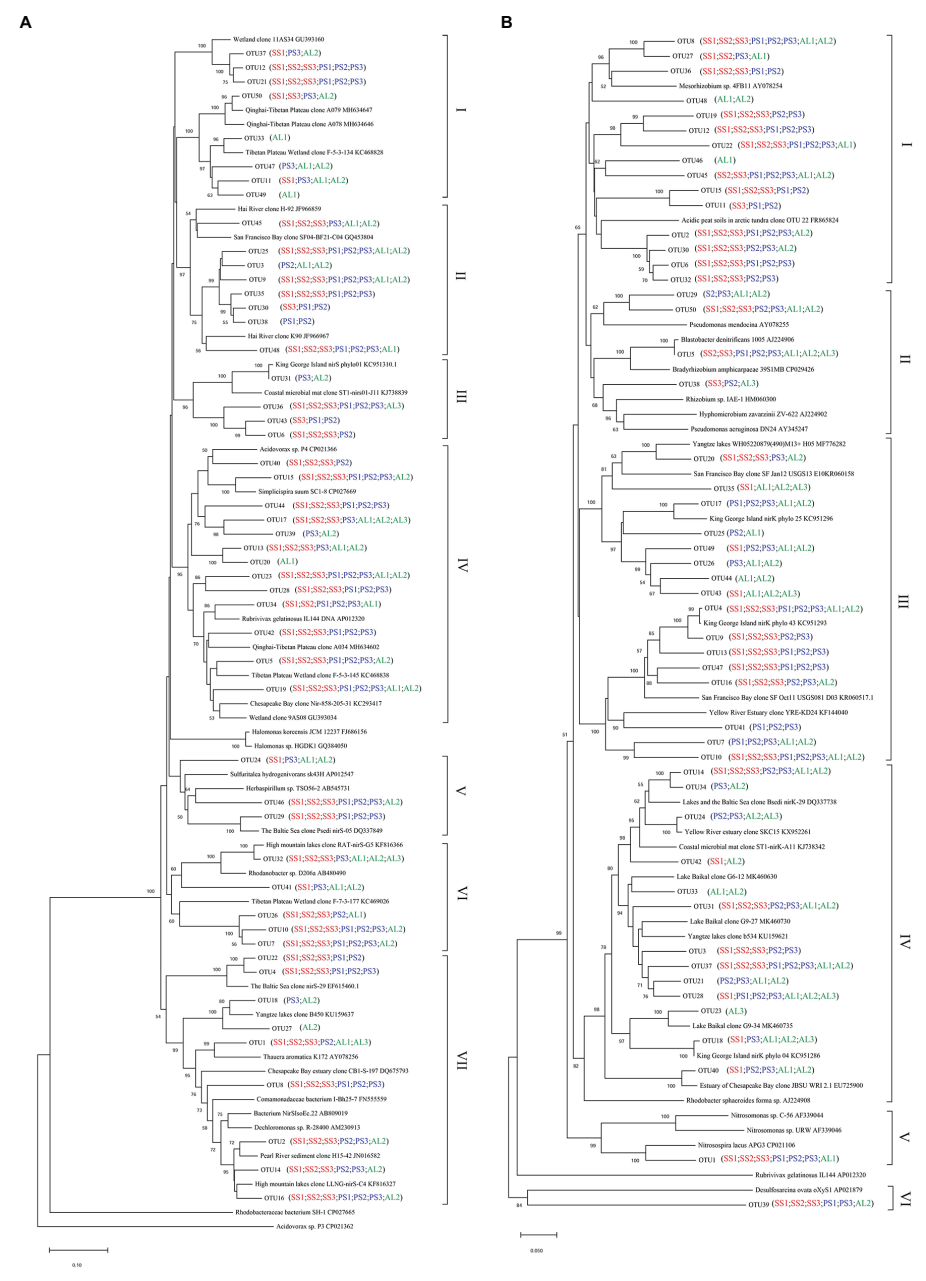
Neighbor-joining phylogenetic tree of nirS **(A)** and nirK **(B)** sequences in tundra soils of maritime Antarctica. Bootstrap values ≥ 50% (*n* = 1,000) are shown near the nodes. Operational taxonomic units (OTUs) were defined at 97% similarity.

**Figure 7:**
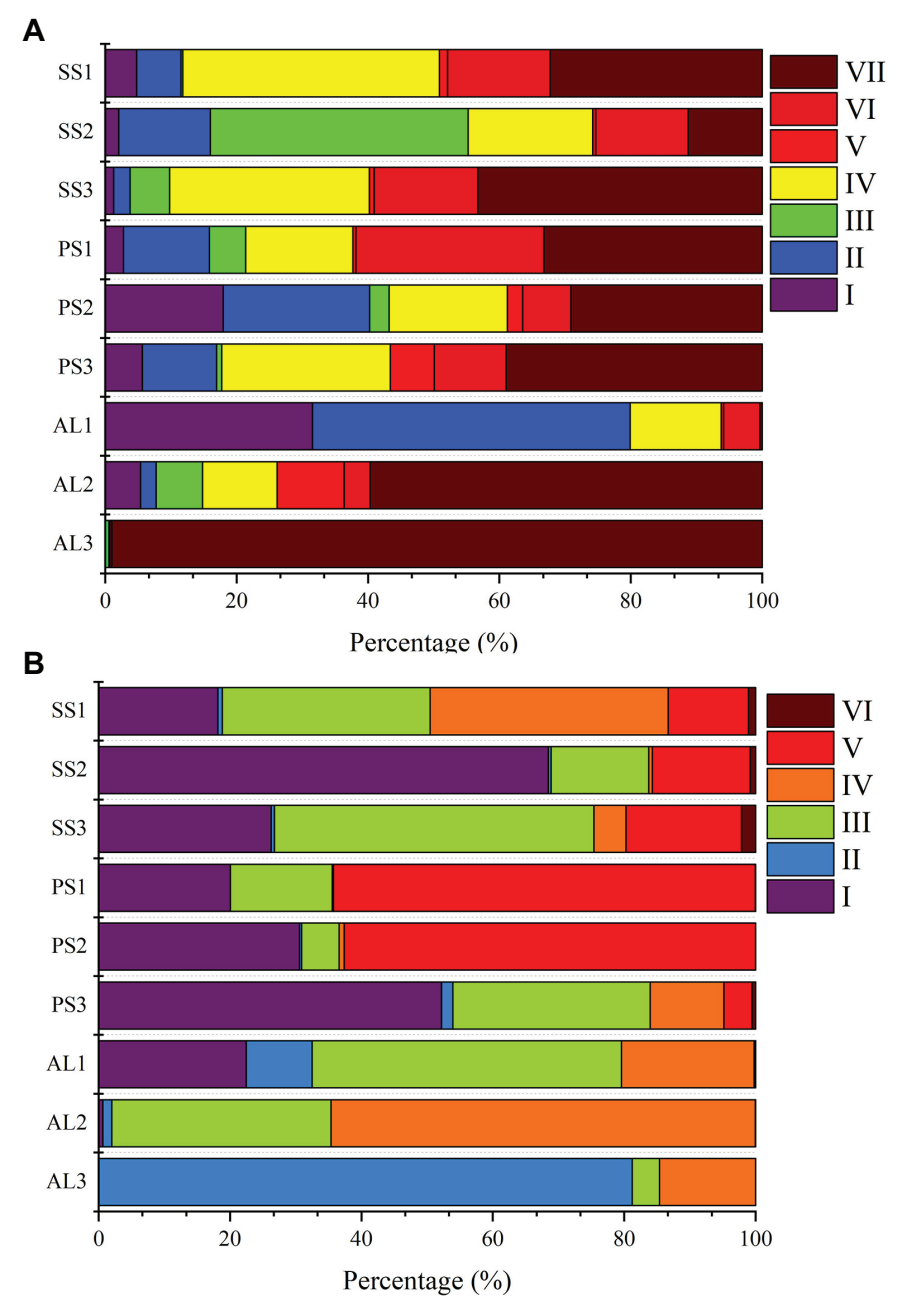
Relative abundances of nirS-encoding **(A)** and nirK-encoding **(B)** denitrifiers sequences retrieved from penguin and seal colony soils and animal-lacking tundra soils, as related to different nirS-encoding and nirK-encoding denitrifier clusters. The analysis was based on the most abundant 50 OTUs from different tundra soils of maritime Antarctica.

Phylogenetic analysis of nirS genes showed that a large proportion of unique OTUs matched to uncultured environmental nirS assemblages (Figure 6A). The majority of tundra soil nirS OTUs found in maritime Antarctica, closely matching to the sequences in the Genbank, were obtained from some environments but not always from obviously similar environments. The minor nirS OTUs matching to bacteria were assigned to *Proteobacteria* in tundra soils. The most abundant 50 OTUs were grouped into distinctly defined seven clusters (I–VII) on the basis of evolutionary distance. The cluster I contained eight OTUs, and 31,340 nirS gene sequences with 53.3% of the sequences from AL, 36.5% from PS, and 10.2% from SS, and they were closely related to those in sediments of Qinghai-Tibetan Plateau river (MH634647) and Tibetan Plateau wetland (KC468828). The cluster II contained eight OTUs, and 51,450 sequences with 45.8% of the sequences from AL, 35.6% from PS, and 18.6% from SS, and they closely matched to those in estuarine sediments from San Francisco Bay (GQ453804) (Mosier and Francis, 2010). The cluster III contained four OTUs and 25,099 sequences with 77.7% from SS, 11.6% from PS, and only 10.7% from AL, and their sequences were closely related to those found in the microbial mats of King George Island (KC951310) (Alcántara-Hernández et al., 2014). The cluster IV contained 13 OTUs and 69,288 sequences, and 50.5% of sequences were from SS, 34.2% from PS, and only 15.3% from AL. The cluster V contained three OTUs and 8,986 sequences, only accounting for 2.5%, and phylogenetically similar to *Proteobacteria*, with 47.2% of sequences from PS, 42.2% from AL, and 10.6% from SS. The cluster VI contained five OTUs and 37,097 sequences, and 39.8% of sequences were from PS, 49.3% from SS, and only 10.9% from AL. The cluster VII had nine OTUs and the most nirS gene sequences (130,157), accounting for 36.8% of the total 50 OTU sequences, with 43.9% from AL, 29.7% PS, and 26.4% from SS, and they closely matched to those found in Baltic Sea (EF615460.1), Yangtze lakes (KU159637), and Pearl River (JN016582). Overall, tundra soils contained all the seven clusters except AL1 and AL3. The clusters III, IV, VI, and VII appeared in all tundra soils. The dominant clusters in nirS genes from penguin and seal colony soils were II, IV, VI, and VII, accounting for 58.3–92.0% of nirS gene sequences (Figure 7A). The dominant clusters in AL1 were clusters I and II, accounting for 79.8%. As for AL2 and AL3, the most dominant cluster was cluster VII, accounting for 59.7 and 99.0%, respectively. The nirS gene sequences of AL3 had only one OTU (OTU1) in cluster VII, which was aligned with *Pseudomonas stutzeri* SLG510A3-8 (CP011854) and had 100.0% identity from the Genbank.

Similar to nirS genes, phylogenetic analysis of nirK genes also showed that a large proportion of unique OTUs matched to uncultured environmental nirK assemblages. The most abundant 50 nirK OTUs were grouped into six distinctly defined clusters (I–VI) on the basis of evolutionary distance (Figure 6B). The most abundant cluster I contained 15 OTUs and 78,457 sequences, accounting for 29.0% of total 50 OTU sequences, with 44.1% from PS, 50.5% from SS, and only 5.4% from AL, and they were closely related to *Mesorhizobium* sp. (AY078254) (Song and Ward, 2006) and similar environment sequences from acidic peat soils in arctic tundra (FR865824) (Palmer et al., 2012). The cluster II contained four OTUs and 17,862 sequences with 3.7% of sequences from PS, 3.1% from SS, and 93.2% from AL. The cluster III contained 16 OTUs and 66,761 sequences with 24.0% from PS, 49.8% from SS, and 26.2% from AL, whereas the cluster IV contained 13 OTUs and 42,125 sequences with 8.7% from PS, 39.2% from SS, and 52.1% from AL. These two clusters included 24.7 and 15.6% of total sequences, respectively, and were closely related to those from some lake or estuarine sediments, such as Yangtze lakes (MF776282; KU159621) (Jiang et al., 2017), San Francisco Bay estuary (KR060158; KR060517) (Lee and Francis, 2017), Yellow River Estuary (KF144040; KX952261) (Li et al., 2014), Lake Baikal (MK460630), Chesapeake Bay estuary (EU725900) (Fortunato et al., 2009), and microbial mats on King George Island (KC951296; KC951293; KC951286) (Alcántara-Hernández et al., 2014). The cluster V contained one OTU and 53,453 sequences with 75.2% from AL, 24.7% from SS, and 0.1% from PS, and cluster VI also contained one OTU and 1,578 sequences with 88.7% from SS, 10.9% from AL, and 0.4% from PS. These two clusters accounted for 23.5 and 0.6% of total sequences, respectively, and were related to *Nitrosospira* (CP021106) (Urakawa et al., 2015) and *Desulfosarcina* (AP021879), respectively. Overall, the clusters II, III, and IV were found in all the nine tundra soils. The dominant clusters from penguin and seal colony soils were I, III, and V, accounting for 62.0 to 99.7% of total sequences (Figure 7B). Clusters III and IV were the dominant clusters for tundra soils AL1 and AL2, respectively, whereas the dominant cluster was II in tundra soils AL3, accounting for 81.2%.

### Relationships of Denitrifier Community Structure With Environmental Variables in Tundra Soils

**Figure 8:**
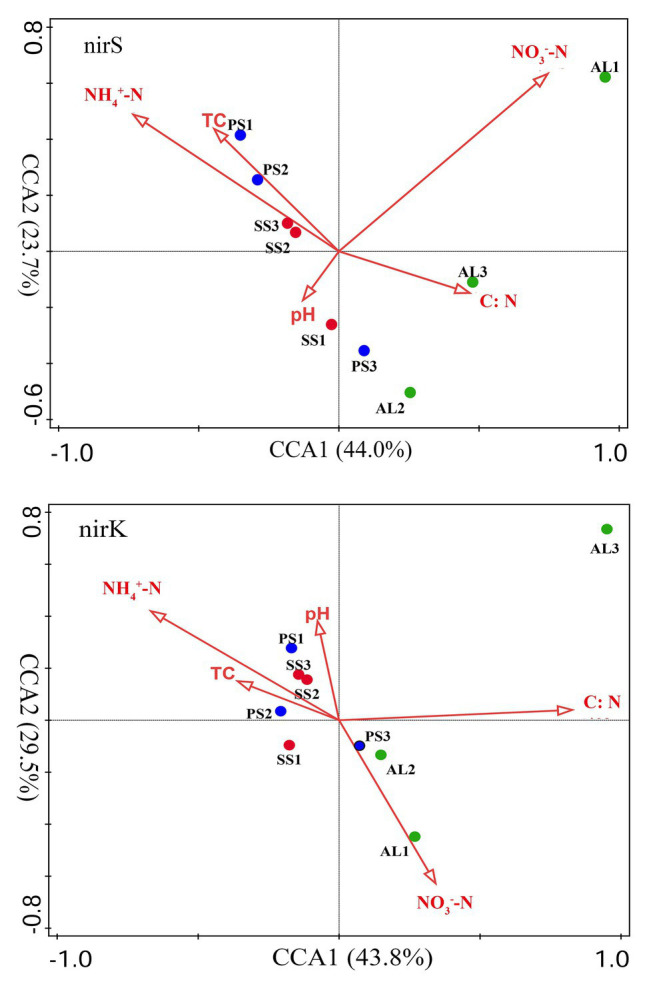
Canonical correspondence analysis (CCA) ordination plots for the relationship between the nirS and nirK community structures with environmental variables. C:N, TC, NH_4_^+^-N, NO_3_^−^-N represent C:N ratios, total carbon, ammonium, and nitrate, respectively.

The relationships of the nirS‐ and nirK-encoding denitrifier community structure with environmental variables were analyzed by the CCA. The environmental variables in the first two CCA dimensions (CCA1 and CCA2) provided 67.7% of cumulative variation of the nirS-encoding denitrifying community-environment relationship (Figure 8). The nirS community structure significantly correlated with NO_3_^−^-N (*F* = 3.2, *p* = 0.012, 1,000 Monte Carlo permutations) and NH_4_^+^-N (*F* = 2.5, *p* = 0.023), which together explained 53.6% of the variation (Table 2). Although other environmental variables, including pH, C:N, and TC were not statistically significant (*p* > 0.05), these variables additionally explained 40.7% of the variation. The first two dimensions explained 73.3% of the cumulative variation of the nirK-encoding denitrifying community-environment relationship. The nirK community structure significantly correlated with C:N (*F* = 2.7, *p* = 0.011) and NH_4_^+^-N (*F* = 2.2, *p* = 0.025), which together explained 51.3% of the variation. Other environmental variables, including NO_3_^−^-N, pH, and TC, accounted for 43.8% of the variation.

**Table 2:** Individual and combined contributions of soil biogeochemical properties to the nirS and nirK community structures in maritime Antarctica.

Gene	Soil properties	*F*	*p*	Individual contribution
nirS	NO_3_^−^-N	3.2	0.012	31.2%
	NH_4_^+^-N	2.5	0.023	22.4%
	TC	1.5	0.147	17.2%
	C:N	1.3	0.216	15.7%
	pH	0.8	0.634	7.8%
	Combined effect			94.3%
nirK	C:N	2.7	0.011	27.5%
	NH_4_^+^-N	2.2	0.025	23.8%
	NO_3_^−^-N	1.8	0.176	20.8%
	TC	1.0	0.390	12.9%
	pH	0.8	0.631	10.1%
	Combined effect			95.1%

## Discussion

### Effects of Sea Animal Activities on the Abundances of Tundra Soil Denitrifiers

In this study, although nirS‐ and nirK-encoding denitrifiers were detected in all tundra soils, the abundances of nirS-encoding denitrifiers were significantly higher than those of nirK-encoding denitrifiers (Figure 2), which agreed with the results from other environments, such as the Yellow River Estuary (Li et al., 2017), Daya Bay (Shi et al., 2019), San Francisco Bay (Lee and Francis, 2017), and Antarctic King Sejong Station (Jung et al., 2011). The nirS gene abundances (3.8 × 10^6^–2.5 × 10^7^ copies g^−1^) in penguin and seal colony soils were close to those from the soils of King Sejong Station in Antarctica (Jung et al., 2011) (10^7^–10^8^ copies g^−1^ soil), higher than those in Tibet Plateau soils (2.03 × 10^4^–1.28 × 10^6^ copies g^−1^) (Wang S. et al., 2019), but lower than those in Arctic seabird-affected taluses soils (6.2 × 10^8^–1.8 × 10^9^ copies g^−1^) (Hayashi et al., 2018). The nirK gene abundances (7.4 × 10^2^–6.2 × 10^5^ copies g^−1^) were significantly lower compared with nirS gene, consistent with those (10^3^–10^6^ copies g^−1^ soil) in soils from King Sejong Station in Antarcitc (Jung et al., 2011), and Svalbard (Hayashi et al., 2018). In addition, both the abundances of nirS and nirK genes were two to four orders of magnitude higher in penguin or seal colony soils than in animal-lacking tundra soils, indicating that sea animal activities greatly increased tundra soil nirS and nirK gene abundances, which were consistent with high bacterial abundances in penguin or seal colony soils and ornithogenic sediments in maritime Antarctica (Ma et al., 2013; Zhu et al., 2015a) and high denitrifier genes in seabird-affected taluses in High Arctic (Hayashi et al., 2018). The nirS gene abundances accounted for only 0.20–0.25% of the bacterial abundances (1.88 × 10^9^–1.00 × 10^10^ gene copies g^−1^) in penguin and seal colony soils, whereas the proportion of nirK gene abundances in the bacterial abundances were two to three orders of magnitude lower than that of nirS gene abundances (Ma et al., 2013).

Our previous studies showed that penguin or seal activities led to generally low C:N ratios, which had been used as an indicator for the intensity of penguin or seal activities in maritime Antarctica (Wang Q. et al., 2019). The abundances of nitrifiers and denitrifiers in soils were significantly correlated with C:N ratios in previous study (Fu et al., 2016). The negative correlations of the lg values of nirS and nirK gene abundances with C:N ratios (Figure 3) indicated that sea animal activities, which changed tundra soil C:N ratios, had an important effect on the denitrifier abundances in tundra soils. Many environmental variables (e.g., temperature, pH, salinity, dissolved oxygen, organic matter, and dissolved inorganic nitrogen) could affect the abundances of soil nirS and nirK genes (Zheng et al., 2015; Fu et al., 2016; Gao et al., 2016; Li et al., 2019; Shi et al., 2019). However, in this study, nirS and nirK gene abundances were not significantly related with other environmental variables (Supplementary Table S1), possibly due to the low number of soil sample replicates within the same type of tundra soils. Overall, the lg values of nirS and nirK gene abundances showed a significant negative correlation with soil C:N ratios across different types of tundra soils. Therefore, the change in tundra soil C:N ratios, associated with sea animal activities, was the predominant factor affecting the denitrifier abundances in tundra soils of maritime Antarctica.

### Effects of Sea Animal Activities on the Activities of Tundra Soil Denitrifiers

The denitrification rates in animal colony soils (5.78–59.49 μmol N kg^−1^ h^−1^) were significantly higher than those in adjacent animal-lacking tundra soils (0.04–0.93 μmol N kg^−1^ h^−1^) (Figure 2), similar to ammonia oxidation rates measured in tundra soils (Wang Q. et al., 2019), indicating that penguin or seal activities had important effects on tundra soil denitrification rates in maritime Antarctica. In addition, the denitrification rates in animal colony soils were also higher than those measured in Tibet Plateau soils (0.39–2.57 μmol N kg^−1^ h^−1^) (Wang S. et al., 2019), but significantly lower than those in Arctic seabird-affected taluses soils (187.1–348.6 μmol N kg^−1^ h^−1^) (Hayashi et al., 2018), similar to those measured in northern Sweden tundra soils (7.1–114.3 μmol N kg^−1^ h^−1^) (Björk et al., 2007), northeast Finland fen soils (36.4 μmol N kg^−1^ h^−1^) (Palmer et al., 2012), and Russian discontinuous permafrost soils (35.7–55.7 μmol N kg^−1^ h^−1^) (Palmer and Horn, 2015) although they were detected at higher temperature (20°C) in these other environments (Supplementary Table S4). The denitrification rates in animal-lacking tundra soils were similar to those in Tibet Plateau soils (Wang S. et al., 2019), but much lower than those in northern Sweden tundra soils (Björk et al., 2007), northeast Finland fen soils (Palmer et al., 2012), Russian discontinuous permafrost soils (Palmer and Horn, 2015), and Arctic seabird-affected talus soils (Hayashi et al., 2018).

Generally, the denitrification rates were affected by the abundances of nirS-encoding denitrifiers (Mosier and Francis, 2010; Gao et al., 2016). In this study, the denitrification rates were significantly positively correlated with nirS gene abundances (*r* = 0.733, *p* = 0.02). However, no significant statistical correlation was obtained between the denitrification rates and nirK gene abundances, tundra soil physicochemical properties. Therefore, compared to nirK gene abundances and soil physicochemical properties, the abundances of nirS genes better predicted the denitrifier activities in tundra soils of maritime Antarctica. In this study, the animal-lacking tundra areas were covered with the cushions of mosses and lichens due to moderate amount of nutrients and the absence of animal trampling. These tundra plants can absorb the limited soil N, highly limit the inorganic N (NH_4_^+^-N) availability for the denitrifiers (Table 1), thus might restrict the denitrification rates in tundra soils (Marushchak et al., 2011; Zhu et al., 2013). In addition, the positive relationship between denitrification rates and temperature had been reported by Hou et al. (2015) and Cheng et al. (2016), which was likely due to the increase in the denitrifier activity (Wallenstein et al., 2006). The denitrifiers are sensitive to temperature with the optimal temperature of 25–27°C for denitrification (Canion et al., 2014), implying that the denitrification rates in tundra soils will be likely to further increase in the context of global warming under the disturbance of penguin or seal activities in maritime Antarctica.

### Effects of Sea Animal Activities on the Diversity of Tundra Soil Denitrifiers

Biological diversity metrics are based on species counts, which require DNA sequence data to be clustered into taxonomic units. Different OTU cutoffs of DNA sequence identity generated different OTU number and Chao 1 index (Lee and Francis, 2017). The OTU number and Chao1 of nirK-encoding denitrifiers were higher than those of nirS-encoding denitrifiers except sites PS2 and AL3. For nirS and nirK, different OTU cutoffs generated little difference in diversity, and all clustering levels led to similar conclusions about the environmental factors influencing denitrifier community compositions (Lee and Francis, 2017). For nirS‐ and nirK-encoding denitrifiers, the Pielou’s Evenness Index and Shannon-Wiener Index (*H*) showed a low variability within the same sample types (Figure 4). However, the Simpson Index (1/*D*) showed a heterogeneous distribution pattern, possibly because the inverse of Simpson Index (1/*D*) is sensitive to the level of dominance in a community (Magurran, 1988). Compared with animal-lacking tundra soils, Shannon and Simpson indexes of nirS-encoding denitrifiers were higher in penguin or seal colony soils (Figure 4), indicating that sea animal activities influenced the nirS-encoding denitrifier diversity in tundra soils. Generally, penguin or seal activities led to generally low C:N ratios, compared to normal tundra soils in maritime Antarctica (Wang Q. et al., 2019). The significant negative correlation (*R* = −0.68, *p* = 0.04) between Pielou’s evenness of nirS-encoding denitrifiers and tundra soil C:N ratios (Figure 5) further indicated a relationship between nirS-encoding denitrifier diversity and sea animal activities.

For nirK-encoding denitrifiers, their diversity was lower than nirS-encoding denitrifiers in sea animal colonies, which was in accordance with other environments, such as sediments of San Francisco Bay (Lee and Francis, 2017), Yangtze Estuary (Zheng et al., 2015), and acidic peat soil in arctic tundra (Palmer et al., 2012). However, in animal-lacking tundra soils (AL1, AL2, and AL3), the diversity of nirK-encoding denitrifiers was higher than that of nirS-encoding denitrifiers (Figure 4). Desnues et al. (2007) found that nirS-encoding denitrifiers were mainly located in the permanent anoxic layer, whereas nirK-encoding denitrifiers were found in zones with high oxygen and pH fluctuations. Animal-lacking tundra soils were covered with cushions of mosses and lichens, which were vertically layered communities with high photosynthetic production rates (Stal, 2012), whereas animal colony soils were devoid of the coverage of tundra vegetation due to toxic overmanuring and trampling (Supplementary Figure S1) (Zhu et al., 2013). Compared with animal colony soils, oxygen concentration fluctuated in animal-lacking tundra soils because of the photosynthesis of mosses or lichens (Fernández-Valiente et al., 2007). Therefore, nirK-encoding denitrifiers more adapted to animal-lacking tundra soils covered with vegetation, compared with animal colony soils. Alcántara-Hernández et al. (2014) also reported that the diversity of nirK-encoding denitrifiers was greater than that of nirS-encoding denitrifiers in microbial mats on King George Island of maritime Antarctica. In addition, significant negative relationship of the Pielou’s evenness of nirK genes with TN, TC, and TS levels (Figure 5), further indicated that animal activities probably decreased the nirK-encoding denitrifier diversity in tundra soils of maritime Antarctica. However, there was no significant relationship between Shannon-Wiener Index (*H*) and Simpson Index (1/*D*) for the denitrifiers and soil environmental variables, which could be due to the low number of soil sample replication within the same tundra types.

### Effects of Sea Animal Activities on Compositions of Tundra Soil Denitrifiers

Our previous studies showed that the dominant bacterial phyla in animal colony and tundra soils in Antarctica were *Proteobacteria* (mean 48.6%), *Actinobacteria* (mean 16.3%), and *Bacteroidetes* (mean 8.6%), and *Proteobacteria* was the most dominant bacterial phylum (Ma et al., 2013). However, in this study, the minor nirS and nirK OTUs matching to bacteria were assigned to *Proteobacteria*. Phylogenetic analysis of nirS and nirK genes showed that a large proportion of OTUs matched to uncultured environmental assemblages (Figure 6). In addition, the nirS‐ and nirK-encoding denitrifier compositions were similar among the soils in penguin or seal colony, but they were different among animal-lacking tundra soils. Within all seven clusters in nirS genes, clusters II, IV, VI, and VII were the dominant phylotypes of animal colony soils, while the dominant phylotypes of AL2 and AL3 was only cluster VII. As for nirK genes, the dominant phylotypes in animal colony soils were clusters I, III, and V, whereas clusters II, III, and IV were dominant phylotypes in animal-lacking tundra soils. This result indicated that the denitrifier community structure in tundra soils was affected by sea animal activities, similar to tundra soil AOA and AOB in maritime Antarctica (Wang Q. et al., 2019). Nitrate and ammonium concentrations, and C:N ratios were closely related to sea animal activities in coastal Antarctica (Riddick et al., 2012; Zhu et al., 2013, 2015b; Otero et al., 2018). Significant correlations between the nirS-encoding denitrifier community structures and NO_3_^−^ and NH_4_^+^-N concentrations, and between nirK-encoding denitrifier community structures and C:N ratios and NH_4_^+^-N concentrations, further confirmed that sea animal activity had an important effect on the denitrifier community structure in tundra soils of maritime Antarctica (Figure 8 and Table 2).

Generally, the nutrient contents are low in normal tundra soils of maritime Antarctica. Sea animals provided considerable external N inputs for their colony soils through direct input of their excreta and ammonia volatilization (Zhu et al., 2011; Riddick et al., 2012). The uric acid from penguin guano or seal excreta produced NH_3_ or NH_4_^+^ through mineralization and ammonification, which increased the nutrient availability in soils (Kuypers et al., 2018; Otero et al., 2018). As summarized in Table 1, ammonium concentrations were one to two orders of magnitude higher in penguin or seal colony soils than in animal-lacking tundra soils. Ammonium levels were one of important environmental parameters affecting the denitrifier community compositions (Figure 8 and Table 2), and it could be oxidized to nitrate by nitrification, and bacteria respired nitrate as a substitute terminal electron acceptor for denitrification (Cornwell et al., 2014). Our previous studies indicated that ammonia oxidation rates were significantly higher in animal colony soils (mean 70.4 μg N kg^−1^ h^−1^) than in non-animal tundra soils. Therefore, the NO_3_^−^ from ammonium nitrification was likely important substrate for soil denitrification in sea animal colony (Wang Q. et al., 2019). At the same time, denitrification rates were also greatly higher in sea animal colony soils than in animal-lacking tundra soils (Figure 2), which might greatly consume considerable nitrate in tundra soils, and lead to low nitrate levels in animal colony soils, even closed to or lower than those in animal-lacking tundra soils (Table 1). The community structures of nirS-encoding denitrifiers had significant correlation with nitrate and ammonium concentrations in tundra soils (Figure 8), which was consistent with previous studies (Zheng et al., 2015; Gao et al., 2016; Lee and Francis, 2017; Shi et al., 2019).

In addition, the community compositions of nirK-encoding denitrifiers were also significantly affected by ammonium. The C:N ratios were also found to be important environmental parameters influencing the community structure of nirK-encoding denitrifiers, which was consistent with the results from different environments (Saleh-Lakha et al., 2009; Mosier and Francis, 2010; Li et al., 2019). Soil pH was an important factor controlling the diversity and composition of denitrifying communities in the environments (Saleh-Lakha et al., 2009; Wise et al., 2019). However, there were no significant correlation between pH and denitrifier diversity, nirS-encoding or nirK-encoding denitrifier community structures in tundra soils (Supplementary Table S3), similar to the results in Tibetan wetlands reported by Jiang et al. (2020). Other environmental factors, such as organic carbon and nitrite, had no significant contribution to nirS and nirK-encoding denirifiers community structures in this study, although they had been reported to have significant impact on denitrifying community compositions (Mosier and Francis, 2010; Francis et al., 2013). Therefore, the community structures of nirS‐ and nirK-encoding denirifiers were closely related to the biogeochemical factors associated with sea animal activities, such as the supply of nitrate and ammonium from sea animal excreta and low C:N ratios, in tundra soils of maritime Antarctica.

## Conclusion

This study provided a comprehensive insight into abundance, activity, diversity, and composition of nirS‐ and nirK-encoding denitrifier communities in tundra soils of maritime Antarctica. The abundances of nirS genes were significantly higher than those of nirK genes in all tundra soils. The denitrification rates in sea animal colony soils were significantly higher than those in adjacent animal-lacking tundra soils, and they were significantly correlated with nirS gene abundances, instead of nirK gene abundances. The lg values of nirS and nirK gene abundances were significantly negatively correlated with tundra soil C:N ratios, indicating that sea animal activities had an important effect on the abundances of tundra soil denitrifiers. The diversity for nirS-encoding denitrifier community was higher in seal or penguin colony soils than in adjacent animal-lacking tundra soils, but the diversity for nirK-encoding denitrifier was lower. The nirS‐ and nirK-encoding denitrifier community structures were influenced by soil biogeochemical processes related to marine animal activities, such as soil C:N alteration, the supply of NH_4_^+^-N and NO_3_^−^-N from animal excreta. This study contributes to understand soil denitrifier communities in tundra environment of maritime Antarctica.

## Data Availability Statement

The datasets presented in this study can be found in online repositories. The names of the repository/repositories and accession number(s) can be found in the article/Supplementary Material.

## Author Contributions

R-BZ, H-TD, and B-WS designed the experiments. L-JH provided experiments platform. H-TD, B-WS, and C-SC performed the experiments and analyzed the data. H-TD and R-BZ prepared original draft. R-BZ and H-TD reviewed and edited the manuscript. All authors contributed to the manuscript and gave final approval for publication.

### Conflict of Interest

The authors declare that the research was conducted in the absence of any commercial or financial relationships that could be construed as a potential conflict of interest.

## Footnotes

^1^http://chinare.mnr.gov.cn/catalog/meteorological
