# Supplementary material for: Effects of Sea Animal Activities on Tundra Soil Denitrification and nirS‐ and nirK-Encoding Denitrifier Community in Maritime Antarctica
Source: Front Microbiol. 2020 Oct 9;11:573302. doi: 10.3389/fmicb.2020.573302 (PMC7581892; doi:10.3389/fmicb.2020.573302)
Supplement: Supplementary file 1 [file Data_Sheet_1.docx]

**Supplementary Material**

**Effects of sea animal activities on tundra soil denitrification and nirS- and nirK-encoding denitrifier community in maritime Antarctica**

Hai-Tao Dai^1^, Ren-Bin Zhu^1^*, Bo-Wen Sun^1^, Chen-Shuai Che^1^ and Li-Jun Hou^2^

^1^Anhui Province Key Laboratory of Polar Environment and Global Change, School of Earth and Space Sciences, University of Science and Technology of China, Hefei 230026, China.

^2^State Key Laboratory of Estuarine and Coastal Research, East China Normal University, Shanghai 200062, China

*Corresponding author: Email: zhurb@ustc.edu.cn; Tel: 0086-551-3606010; Fax: 0086-551-63606010.


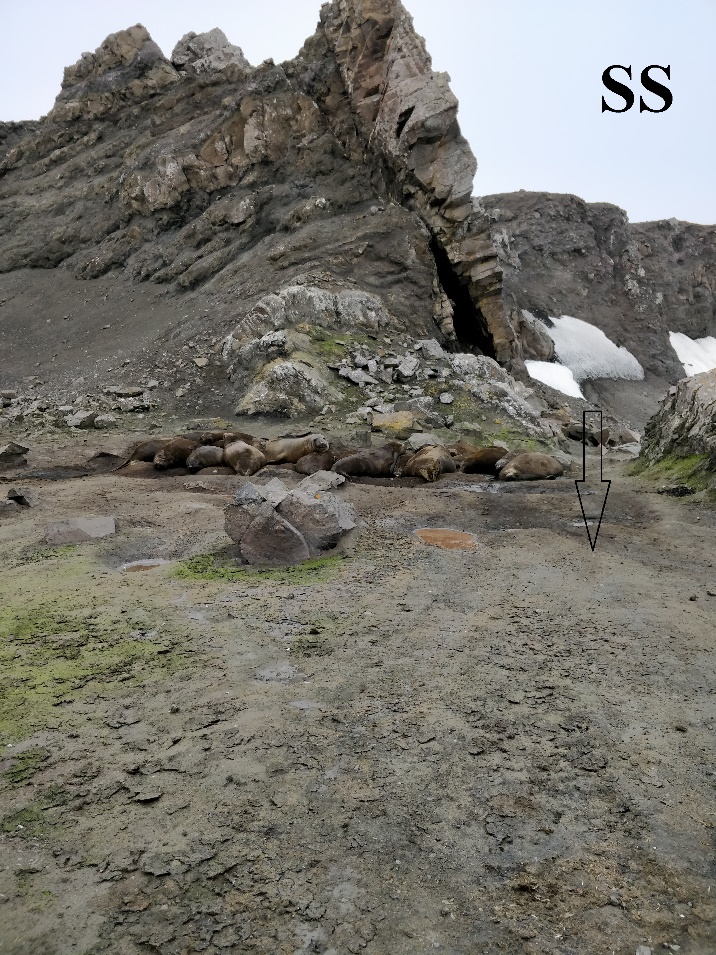

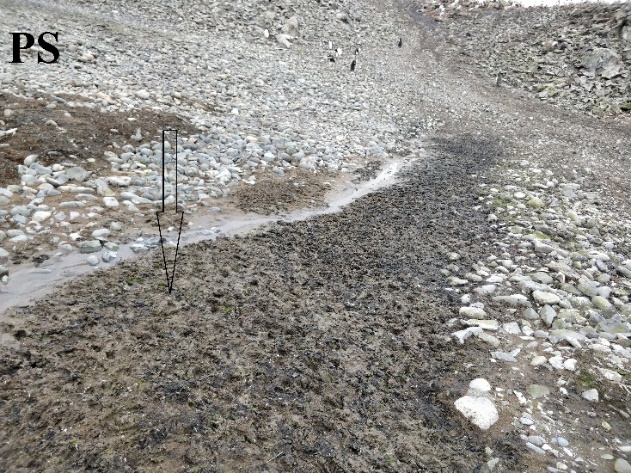


**
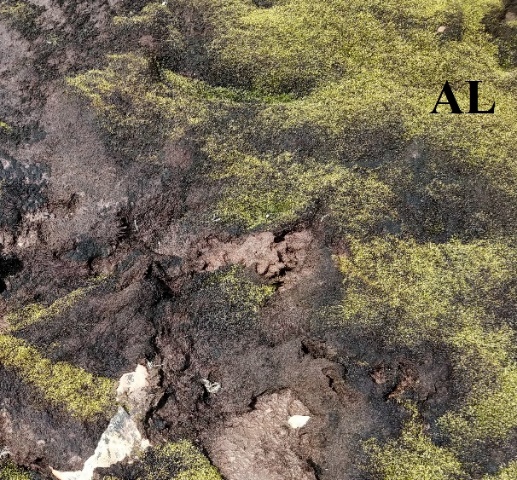
**

**Figure S1.** Sampling points in the maritime Antarctica. PS (PS1–3) were collected in the eastern active penguin colony, showing penguin tramping and the deposition of penguin guano. SS (SS1–3) were collected in the western seal colony, showing seal hairs and the deposition of seal excreta. The arrow indicated the sampling area. AL (AL1–3) were collected in adjacent animal-lacking tundra area covered by cushions of mosses, lichens, and algae due to moderate amount of nutrients and the absence of animal trampling.

**Table S1.** Pearson’s correlation coefficients of nirS and nirK genes abundances, and denitrification rates with tundra soil physicochemical properties (n = 9).

|  |  | pH | MC | OM | TN | TC | TS | TP | C: N | NH_4_^+^ | NO_3_^-^ |
| --- | --- | --- | --- | --- | --- | --- | --- | --- | --- | --- | --- |
| nirS gene Abundance | R | 0.37 | -0.17 | -0.15 | -0.04 | -0.04 | 0.15 | -0.17 | -0.60 | 0.32 | -0.43 |
|  | P | 0.33 | 0.65 | 0.69 | 0.92 | 0.91 | 0.70 | 0.66 | 0.09 | 0.40 | 0.25 |
| nirK gene Abundance | R | 0.10 | -0.11 | -0.20 | -0.17 | -0.13 | -0.05 | -0.20 | -0.28 | -0.14 | -0.27 |
|  | P | 0.80 | 0.77 | 0.60 | 0.67 | 0.74 | 0.90 | 0.61 | 0.47 | 0.72 | 0.48 |
| Denitrification rates | R | 0.42 | -0.19 | -0.22 | -0.05 | -0.12 | 0.17 | -0.17 | -0.50 | 0.41 | -0.30 |
|  | P | 0.26 | 0.62 | 0.57 | 0.91 | 0.75 | 0.66 | 0.67 | 0.18 | 0.27 | 0.43 |

**Table S2.** Richness and diversity characteristics of nirS and nirK genes in tundra soils of maritime Antarctica.

| Gene | Site | OTUs | Chao1 | Pielou’s Evenness  Index | Shannon-Winer  Index [*H*] | Simpson  Index [1/*D*] | Coverage |
| --- | --- | --- | --- | --- | --- | --- | --- |
| nirS | SS1 | 173 | 173 | 0.63 | 3.23 | 11.01 | 1.00 |
|  | SS2 | 135 | 135 | 0.62 | 3.02 | 8.09 | 1.00 |
|  | SS3 | 219 | 219 | 0.67 | 3.59 | 13.54 | 1.00 |
|  | PS1 | 128 | 128 | 0.68 | 3.28 | 12.44 | 1.00 |
|  | PS2 | 299 | 299 | 0.67 | 3.83 | 19.09 | 1.00 |
|  | PS3 | 209 | 209 | 0.72 | 3.83 | 25.68 | 1.00 |
|  | AL1 | 250 | 250 | 0.49 | 2.69 | 5.40 | 1.00 |
|  | AL2 | 304 | 304 | 0.64 | 3.67 | 11.60 | 1.00 |
|  | AL3 | 141 | 141 | 0.37 | 1.85 | 1.98 | 1.00 |
| nirK | SS1 | 229 | 229 | 0.52 | 2.81 | 7.5 | 0.99 |
|  | SS2 | 186 | 186 | 0.58 | 3.04 | 10.03 | 1.00 |
|  | SS3 | 325 | 325 | 0.66 | 3.83 | 23.83 | 1.00 |
|  | PS1 | 307 | 307 | 0.47 | 2.70 | 4.89 | 1.00 |
|  | PS2 | 241 | 241 | 0.44 | 2.39 | 3.38 | 0.99 |
|  | PS3 | 288 | 288 | 0.63 | 3.57 | 14.12 | 1.00 |
|  | AL1 | 458 | 458 | 0.75 | 4.58 | 46.33 | 1.00 |
|  | AL2 | 335 | 335 | 0.71 | 4.14 | 30.71 | 1.00 |
|  | AL3 | 120 | 120 | 0.62 | 2.97 | 5.66 | 1.00 |

| Diversity indexes |  | pH | MC | OM | TN | TC | TS | TP | C: N | NH_4_^+^ | NO_3_^-^ |
| --- | --- | --- | --- | --- | --- | --- | --- | --- | --- | --- | --- |
| nirS Chao1 | R | -0.39 | 0.23 | 0.06 | 0.18 | 0.07 | 0.23 | 0.29 | -0.19 | 0.06 | 0.25 |
|  | P | 0.37 | 0.55 | 0.87 | 0.65 | 0.86 | 0.56 | 0.44 | 0.63 | 0.88 | 0.51 |
| nirS Pielou’s evenness | R | -0.29 | 0.43 | 0.38 | 0.49 | 0.49 | 0.59 | 0.46 | **-0.68** | 0.56 | -0.36 |
|  | P | 0.45 | 0.25 | 0.31 | 0.18 | 0.18 | 0.10 | 0.22 | **0.04** | 0.12 | 0.34 |
| nirS Shannon Winer | R | -0.36 | 0.44 | 0.34 | 0.48 | 0.43 | 0.59 | 0.48 | **-0.64** | 0.50 | -0.27 |
|  | P | 0.34 | 0.24 | 0.38 | 0.20 | 0.24 | 0.10 | 0.19 | **0.06** | 0.17 | 0.48 |
| nirS Simpson | R | -0.49 | 0.62 | 0.42 | 0.53 | 0.52 | 0.63 | 0.53 | -0.35 | 0.43 | -0.31 |
|  | P | 0.18 | 0.08 | 0.26 | 0.15 | 0.15 | 0.07 | 0.14 | 0.36 | 0.25 | 0.41 |
| nirK Chao1 | R | -0.43 | 0.27 | 0.11 | 0.03 | 0.10 | -0.06 | 0.13 | -0.25 | -0.12 | 0.67 |
|  | P | 0.25 | 0.49 | 0.79 | 0.95 | 0.80 | 0.88 | 0.74 | 0.51 | 0.76 | 0.06 |
| nirK Pielou’s evenness | R | 0.22 | -0.42 | -0.67 | **-0.73** | **-0.68** | **-0.72** | **-0.63** | 0.20 | -0.63 | 0.44 |
|  | P | 0.56 | 0.27 | 0.051 | **0.03** | **0.04** | **0.03** | **0.07** | 0.61 | 0.07 | 0.23 |
| nirK Shannon Winer | R | 0.04 | -0.24 | -0.49 | -0.56 | -0.50 | -0.56 | -0.45 | 0.02 | -0.51 | 0.56 |
|  | P | 0.93 | 0.54 | 0.18 | 0.12 | 0.17 | 0.11 | 0.22 | 0.95 | 0.15 | 0.12 |
| nirK Simpson | R | -0.05 | -0.17 | -0.38 | -0.43 | -0.40 | -0.46 | -0.32 | -0.06 | -0.42 | 0.48 |
|  | P | 0.89 | 0.66 | 0.31 | 0.25 | 0.28 | 0.22 | 0.40 | 0.87 | 0.26 | 0.19 |

**Table S3.** Pearson’s correlation coefficients between the diversity of nirS and nirK denitrifiers and physicochemical properties in tundra soils of maritime Antarctica (n = 9).

**Table S4.** Comparisons of the denitrification rates in this study with those in other ecosystems.

| Names | Rates (µmol N kg^-1^ h^-1^) | Temperature (℃) | References |
| --- | --- | --- | --- |
| Animal colony soils | 5.78-59.49 | 10 | This study |
| Animal-lacking tundra soils | 0.04-0.93 | 10 | This study |
| Tibet Plateau soils | 0.39- 2.57 | In situ temperature | Wang et al., 2019b |
| Arctic seabird-affected taluses soils | 187.1-348.6 | 10 | Hayashi et al., 2018 |
| Northern Sweden tundra soils | 7.1-114.3 | Room temperature | Björk et al., 2007 |
| Northeast Finland fen soils | 36.4 | 20 | Palmer et al., 2012 |
| Russian discontinuous permafrost soils | 35.7-55.7 | 20 | Palmer et al., 2015 |


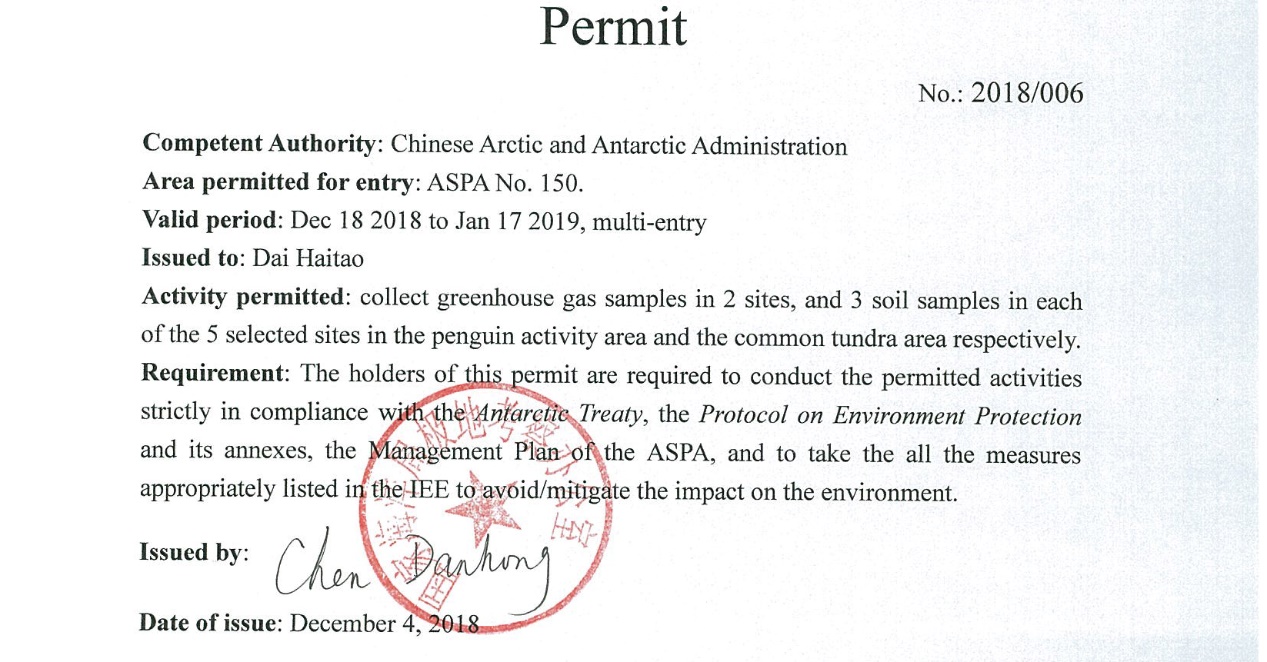


**Figure S2.** The collection permits used for this research
